# First molecular characterization of *Sarcocystis tenella* in Tatra chamois (*Rupicapra rupicapra tatrica*) in Poland

**DOI:** 10.1007/s00436-015-4619-4

**Published:** 2015-07-24

**Authors:** Rafał Kolenda, Peter Schierack, Filip Zieba, Tomasz Zwijacz-Kozica, Michał Bednarski

**Affiliations:** Faculty of Natural Sciences, Brandenburg University of Technology Cottbus-Senftenberg, Großenhainer Str. 57, D-01968 Senftenberg, Germany; Tatra National Park, Kuźnice 1, 34-500 Zakopane, Poland; Department of Epizootiology and Clinic of Bird and Exotic Animals, Wrocław University of Environmental and Life Sciences, 50-375 Wrocław, Poland

**Keywords:** *Sarcocystis tenella*, Tatra chamois, *cox1*, *ssu rRNA*, Phylogenetic analysis

## Abstract

In this study, sarcocysts from three Polish Tatra chamois were isolated and identified using morphological and molecular methods for the first time. Six cysts were found in the latissimus dorsi muscle and another two in the diaphragm. No sarcocysts were detected in the myocardium, tongue, and esophagus. The isolated cysts were long with rounded ends, 0.35–0.61 mm in length, and 0.02–0.06 mm in width. All the sarcocysts were identified as *Sarcocystis tenella* on the basis of light microscopy and sequencing of cytochrome C oxidase subunit I (*cox1*) and small-subunit rRNA (*ssu rRNA*) genes. Comparative analysis showed a 99.23 % identity of the *cox1* gene sequences from Tatra chamois and sheep sarcocysts, and an even higher degree of sequence identity (99.88 %) was documented in the case of the *ssu rRNA* gene. When compared at a haplotype level, all the sheep sequences of *cox1* differed from those isolated from Tatra chamois. In contrast, one out of the two *ssu rRNA* haplotypes from the sheep isolates was identical with the haplotype from Tatra chamois. In conclusion, we showed that *cox1* and *ssu rRNA* genes can be used as genetic markers for identification of the *S. tenella*, with *cox1* gene providing better resolution during phylogenetic analyses. However, both genetic population analysis and phylogenetic inference with *cox1* and *ssu rRNA* genes demonstrated that they do not constitute good markers for spatial differentiation of *S. tenella*.

**Electronic supplementary material:**

The online version of this article (doi:10.1007/s00436-015-4619-4) contains supplementary material, which is available to authorized users.

## Introduction

*Sarcocystis* spp. are protozoan intracellular parasites with a two-host life cycle (Fayer 2004). Their intermediate hosts are herbivores and omnivores, while carnivores constitute the definitive hosts. After ingestion of oocysts by the intermediate host, *Sarcocystis* spp. undergo asexual generations in the vascular endothelial cells in all parts of the body with eventual formation of mature sarcocysts in the hosts’ muscles. The definitive host is infected due to ingestion of the *Sarcocystis*-infected muscular tissue, which results in the intestinal generation of oocysts (Fayer et al. 2015).

The Bovidae family consists of 143 species (Heller et al. 2013), including many well-established intermediate hosts for *Sarcocystis* spp. (Tenter 1995). While *Sarcocystis* spp. found in domesticated Bovidae, e.g., cattle, sheep, and goats, have been characterized extensively (Dubey et al. 1989, 2014; Morsy et al. 2011; Formisano et al. 2013), recent research centers around wild ruminants as intermediate hosts for these protozoa (Dahlgren and Gjerde 2007; Gjerde 2013). However, still little is known about *Sarcocystis* spp. infection in many wild ruminant species, e.g., in Tatra chamois (*Rupicapra rupicapra tatrica*). Tatra chamois is listed as a critically endangered species in the IUCN Red List of Threatened Species (Aulagnier et al. 2008). The animals from this subspecies can be found in Polish and Slovak Tatra mountains. The population of Tatra chamois was estimated at only 220 in 1999 (Jurdíková 2000) and at 950 in 2012 (Tatra National Park Information). Due to such small size of Tatra chamois population, an information about the ecology and pathology of *Sarcocystis* spp. in this species becomes of vital importance. While *Sarcocystis* spp. were demonstrated as a cause of enzootic muscle tissue parasitoses in other ruminants, with significant losses in livestock due to abortions, reduced weight, neurological disease, and even mortality in rarely observed acute cases (Caldow et al. 2000; Schock et al. 2012; Agerholm and Dubey 2014), we still lack a data on the prevalence and pathogenic role of *Sarcocystis* spp. in Tatra chamois.

*Sarcocystis* spp. can be identified with morphological and molecular methods. Molecular techniques (Tenter et al. 1994; Morgan and Thompson 1999) have successfully replaced morphological methods as the latter was shown to be more prone to misjudgment. Cytochrome C oxidase subunit I (*cox1*) and small-subunit rRNA (*ssu rRNA*) gene sequencing was the primary molecular technique used in most recently published studies dealing with the problem in question (Dahlgren and Gjerde 2010; Gjerde 2012). The results of these studies can be used for characterization of inter- and intraspecies phylogenetic relationships. However, the question which of these two genes is more suitable for species determination and phylogenetic analyses is still a subject of ongoing discussion (Gjerde 2013). It is noteworthy that the *ssu rRNA* sequences turned out to be indistinguishable for some species (e.g., *Sarcocystis tarandi* and *Sarcocystis elongata*, *Sarcocystis rangiferi* and *Sarcocystis truncata*). In contrast, the analysis based on *cox1* gene sequences provided satisfactory resolution both on the species level and within the phylogenetic inference (Gjerde 2014a).

To the best of our knowledge, no data on the prevalence of *Sarcocystis* spp. in Tatra chamois have been published to date. Therefore, the aim of this study was to identify *Sarcocystis* spp. present in Tatra chamois and to compare their sequences for *cox1* and *ssu rRNA* genes with those found in other Bovidae. Moreover, our research centered around the phylogenetic placement of the isolated sarcocysts and potential use of *cox1* and *ssu rRNA* genes as molecular markers for phylogeographic studies of newly discovered *Sarcocystis* spp.

## Material and methods

### Isolation and morphological identification of sarcocysts

The study included eight sarcocysts isolated from three adult Tatra chamois individuals found dead in the Polish Tatra National Park during the winter season of 2012 and 2013. The animals did not show any signs of infection and probably died due to multiple injuries caused by avalanches as they presented with broken legs, ribs, and spine. The specimens from the diaphragm and latissimus dorsi muscle (MLD, 200 g), myocardium (30–40 g), tongue (10 g), and esophagus (20–25 g) were examined for the presence of sarcocysts. Individual sarcocysts were excised under a stereomicroscope with an aid of fine needles. A new needle was used for each specimen. Excised sarcocysts were washed three times with PBS and examined under a light microscope (LM). Subsequently, the material was stored at −20 °C for up to 6 months until DNA isolation.

### DNA isolation, amplification of *cox1* and *ssu rRNA* genes, cloning, and sequencing

Total DNA was extracted with a DNeasy Blood & Tissue Kit (QIAGEN GmbH, Germany). The isolation procedure was performed according to the manufacturer’s protocol. The *cox1* and *ssu rRNA* genes were amplified by PCR as described previously, with minor modifications (Kolenda et al. 2014). Briefly, the following primers were used for the amplification of *cox1* and *ssu rRNA* genes: 5′-CGGTATGTACATACTTACGGCG-3′ and 5′-AAACAAATGCAATGGCTGCC-3′ (*cox1*); 5′-GCCATGCATGTCTAAGTATAAGC-3′ and 5′-CCTTGTTACGACTTCTCCTTCC-3′ (*ssu rRNA*). A total of 50 μl of the reaction mix for each primer pair contained DNA (2 μl), Phusion HF buffer (Thermo Scientific), MgCl_2_ (at final concentration of 4 mM), 1 U of Phusion High-Fidelity DNA Polymerase (Thermo Scientific), 0.2 μM of each primer, and 0.2 mM dNTPs mix. PCR was started with initial denaturation (98 °C, 30 s), followed by 40 cycles of denaturation (98 °C, 5 s), annealing (56 °C, 15 s), elongation (72 °C, 1 kb/s), and final elongation (72 °C, 5 min). The PCR products were run on a 1 % agarose gel. Subsequently, the gel was stained with ethidium bromide, and appropriate bands were excised and purified with a MinElute Gel Extraction Kit (QIAGEN, Germany) according to the manufacturer’s protocol. The purified DNA was cloned into the vector pJET 1.2 of the CloneJET PCR Cloning Kit (Thermo Scientific) in line with the manufacturer’s protocol. One *cox1*- and one *ssu rRNA*-positive plasmid from each sarcocyst isolate was sent for sequencing.

### Sequencing, sequence alignment, comparison, and phylogenetic analyses

The *cox1*- and *ssu rRNA*-positive plasmids were sequenced with the following primers: 5′-CGACTCACTATAGGGAGAGCGGC-3′ and 5′-AAGAACATCGATTTTCCATGGCAG-3′ (primer binding sites on the plasmid, used for *cox1* and *ssu rRNA*); 5′-TATCCCCATCACGATGCATAC-3′ (primer binding site on the *ssu rRNA* sequences) (Dahlgren and Gjerde 2007). The sequences were determined by a BigDye® Terminator v3.1 cycle sequencing (Life Technologies) and analyzed on an ABI fluorescence automated DNA sequencer at the LGC Genomics (Berlin, Germany). The sequence reads were assembled into contigs with a CAP3 software (Huang and Madan 1999).

The *cox1* and *ssu rRNA* gene sequences were aligned using the default settings of the ClustalX 2.1 software (Larkin et al. 2007). The alignments for *cox1* and *ssu rRNA* genes, consisting of 375 and 184 sequences, respectively (Table S1), were used for further phylogenetic analyses.

The *cox1* and *ssu rRNA* gene sequences for *Sarcocystis tenella* were compared using an Arlequin 3.5 software (Excoffier and Lischer 2010) and MEGA 6.06 software (Tamura et al. 2013). We analyzed the number of haplotypes (H), number of observed substitutions (S), transitions (Trs) and transversions (Trv), nucleotide diversity (*π*), average number of pairwise differences (Theta-*π*), and mean pairwise distance between the groups/species. The inter-haplotypic distance matrices were generated with an R package (R Development Core Team 2013).

The phylogenetic reconstruction based on a maximum likelihood (ML) and maximum parsimony (MP) was conducted with a MEGA 6.06 software (Tamura et al. 2013). GTR + G + I and K2 + G + I were identified as the optimal substitution models for the *cox1* and *ssu rRNA* ML phylograms, respectively. In the case of the *ssu rRNA* gene, the analyses did not include gaps in alignment. The MP trees for both genes were created with using a subtree-pruning-regrafting (SPR) algorithm. The reliability of the ML and MP phylograms was verified with the bootstrap method. MrBayes v. 3.2 was used for the Bayesian tree construction (Ronquist et al. 2012). The Bayesian searches for the *cox1* gene were initiated with random starting trees based on a GTR + G + I model for 14,000,000 generations, and the tree was sampled every 500 generations. The Bayesian searches for the *ssu rRNA* gene were initiated with random starting trees based on a K2 + G + I model for 10,000,000 generations, and the tree was sampled every 500 generations. The burn-in value for *cox1* and *ssu rRNA* genes was set at 25 %.

## Results

### Isolation and morphological characterization of sarcocysts

The samples from three adult Tatra chamois were examined for the presence of sarcocysts. Six cysts were found in the latissimus dorsi muscle and another two were isolated from the diaphragm specimens (Table 1). We did not find any sarcocysts in the myocardium, tongue, and esophagus. No macroscopic cysts were found. All the isolated cysts were long with rounded ends, 0.35–0.61 mm in length and 0.02–0.06 mm in width. The sarcocysts were not encapsulated by a fibrous material. On light microscopy, the cysts appeared to have smooth (lacking a fibrillary structure) thin walls without any protrusions (Fig. S1).

**Table 1:** Morphological characterization of *Sarcocystis tenella* isolated from Tatra chamois and the GenBank accession numbers for *cox1* and *ssu rRNA* genes

Animal number/sex	Time of isolation	Muscle	Size [mm]	GenBank accession nos.	
				*cox1*	*ssu rRNA*
1/female	March 2012	MLD	0.35 × 0.02	KP263746	KP263754
			0.42 × 0.03	KP263747	KP263755
		Diaphragm	0.58 × 0.05	KP263748	KP263756
2/male	October 2012	MLD	0.61 × 0.05	KP263749	KP263757
		Diaphragm	0.46 × 0.04	KP263750	KP263758
		MLD	0.39 × 0.03	KP263744	KP263752
			0.44 × 0.03	KP263745	KP263753
3/male	February 2013	MLD	0.53 × 0.06	KP263751	KP263759

### Molecular characterization of the *cox1* gene

We obtained eight sequences and compared them with the sequences stored in the GenBank. All the sequences were eventually identified as *S. tenella* and submitted to the GenBank under the accession numbers KP263744–KP263751.

Subsequently, we compared our *cox1* sequences from Tatra chamois with the available sequences of *S. tenella cox1* gene (GenBank Accession No. KC209723–KC209732). All the sequences stored in the GenBank originated from sheep sarcocysts. We found 12 polymorphic sites in the eight *cox1* sequences from Tatra chamois and 33 polymorphic sites in the ten available *cox1* sequences from sheep (Table S2). Compared to the sheep sequences, the sequences from Tatra chamois showed nearly twofold lower nucleotide diversity and average number of pairwise differences per sequence (Table 2). Moreover, the number of private substitutions in the sheep sarcocysts was 3.5-folds higher than in the sarcocysts from Tatra chamois. When the sequences were compared at a haplotype level, all the sheep haplotypes of *Sarcocystis* differed from the Tatra chamois haplotypes (Fig. S2). While all the sheep haplotypes were different, four sequences from Tatra chamois belonged to two haplotypes.

**Table 2:** Molecular diversity of *cox1* and *ssu rRNA* genes from *S. tenella*

	Tatra chamois		Sheep		Together	
	*cox1*	*ssu rRNA*	*cox1*	*ssu rRNA*	*cox1*	*ssu rRNA*
N	8	8	10	4	18	12
H	6	7	10	2	16	8
P	12	10	33	1	41	10
S	12	10	33	1	41	10
Trs	10	10	21	1	28	10
Trv	2	0	12	0	13	0
pS	8	9	29	0	–	–
*π* (±SD)	0.0048 (±0.003)	0.002 (±0.0013)	0.009 (±0.0051)	0.0003 (±0.0003)	0.0075 (±0.0041)	0.0014 (±0.0009)
Theta-*π* (±SD)	4.75 (±2.60)	3.54 (±2.01)	8.87 (±4.47)	0.50 (±0.52)	7.44 (±3.65)	2.61 (±1.50)

*N* number of isolates, *H* number of haplotypes, *P* number of polymorphic sites, *S* number of substitution sites, *Trs* number of transitions, *Trv* number of transversions, *pS* private substitutions sites, *π* nucleotide diversity, *Theta-π* average number of pairwise differences

**Fig. 1:**
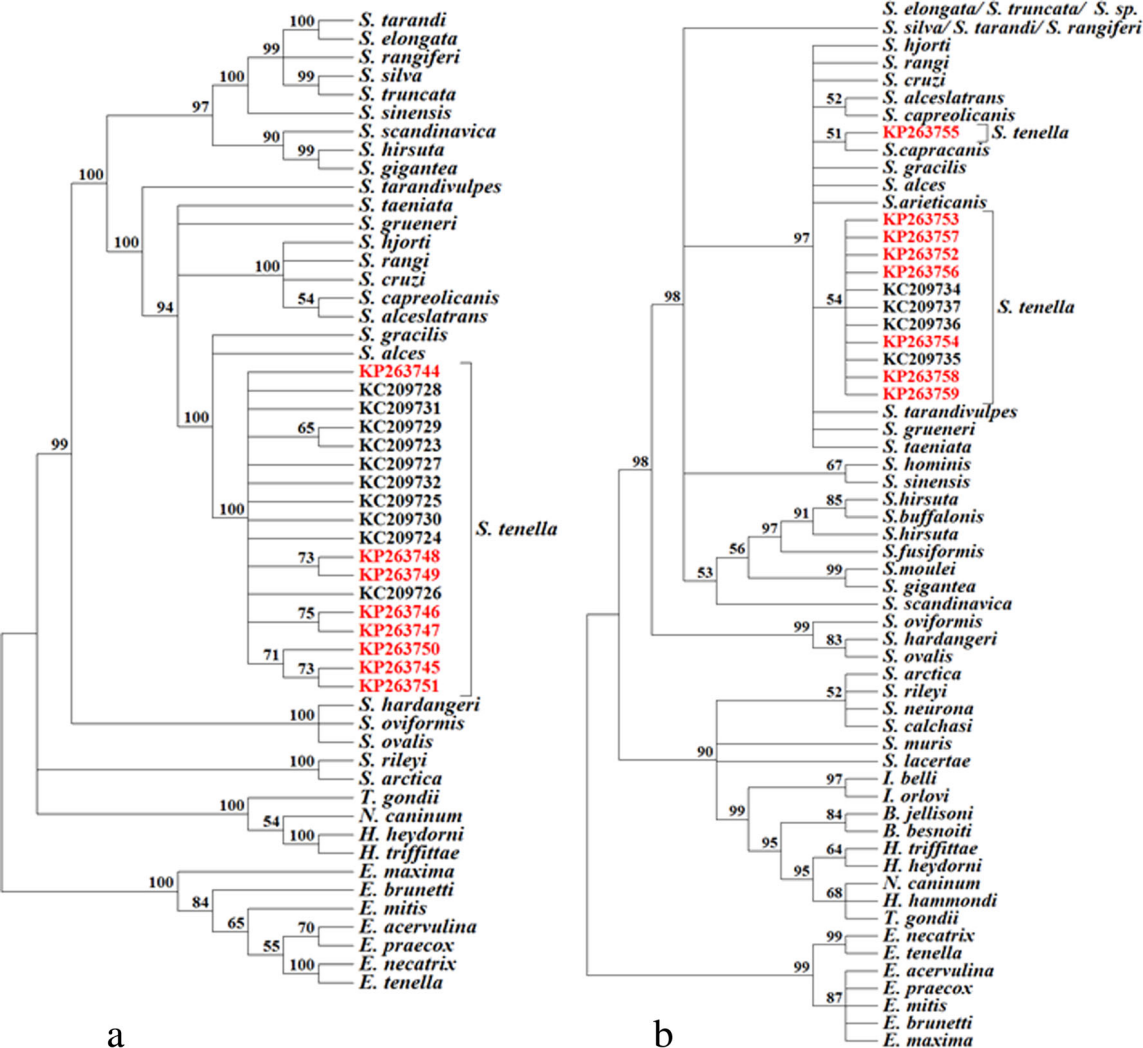
Maximum likelihood for DNA phylograms of selected Sarcocystidae and Coccidia. **a** The *cox1* tree was constructed based on an alignment of partial *cox1* gene sequences from eight Polish *Sarcocystis tenella* isolates and available *cox1* gene sequences of related species deposited in the GenBank (a total of 375 sequences). **b** The ssu rRNA tree was constructed based on an alignment of nearly complete *ssu rRNA* gene sequences from eight Polish *S. tenella* isolates and available *ssu rRNA* gene sequences of related species deposited in the GenBank (a total 184 sequences). The trees were rooted with *Eimeria* spp. The values between the branches represent percent bootstrap value per 1000 replicates. Bootstrap values below 50 % are not shown. Polish isolates are marked in *red*. The GenBank accession numbers of all sequences used for construction of the trees are given in Table S1

The phylogenetic analyses of the *cox1* gene sequences from *Sarcocystis* spp. isolated from cattle, cervids, and sheep were conducted with three methods: ML, MP, and Bayesian inference. As the trees with highly similar clade arrangements were obtained irrespective of the method used, we present only the ML tree (Fig. 1). All the Polish *cox1* sequences from *S. tenella* are placed alongside the sequences from sheep in one cluster. All the sequences from Tatra chamois can be found in separate nodes of this cluster, but they do not tend to group in a single subclade. These findings are further supported by low pairwise distance values between the groups, as shown in Table 3. The Norwegian isolates showed higher intragroup pairwise distance (0.009 ± 0.002) as compared to the intergroup pairwise distance between the Polish and Norwegian isolates (0.008 ± 0.002).

**Table 3:** Comparison of mean pairwise distances for *cox1* sequences (lower block) and *ssu rRNA* sequences (upper block) from selected *Sarcocystis* spp.

	*S. tenella*_P	*S. tenella*_N	*S. gracilis*	*S. alces*	*S. grueneri*	*S. alceslatrans*	*S. rangi*	*S. hjorti*	*S. cruzi*	*S. capreolicanis*	*S. taeniata*	*S. tarandivulpes*	*S. capracanis*	*S .arieticanis*
*S. tenella*_P		0.0007	0.0144	0.0108	0.0162	0.0189	0.0213	0.0197	0.0182	0.0213	0.0202	0.0181	0.0053	0.0283
*S. tenella*_N	0.0076		0.0138	0.0102	0.0163	0.0187	0.0211	0.0193	0.0181	0.0211	0.0197	0.0181	0.0048	0.0283
*S. gracilis*	0.1207	0.1215		0.0118	0.0211	0.0253	0.0265	0.0235	0.0247	0.0255	0.0249	0.0223	0.0157	0.0331
*S. alces*	0.1266	0.1276	0.1155		0.0197	0.0223	0.0241	0.0223	0.0223	0.0249	0.0220	0.0227	0.0132	0.0301
*S. grueneri*	0.1821	0.1827	0.1706	0.1715		0.0241	0.0259	0.0259	0.0241	0.0269	0.0187	0.0223	0.0205	0.0313
*S. alceslatrans*	0.1837	0.1807	0.1821	0.1791	0.1752		0.0096	0.0096	0.0070	0.0078	0.0215	0.0253	0.0229	0.0259
*S. rangi*	0.1891	0.1871	0.1852	0.1771	0.1690	0.1094		0.0084	0.0078	0.0100	0.0208	0.0289	0.0253	0.0295
*S. hjorti*	0.1886	0.1867	0.1718	0.1767	0.1719	0.0932	0.0846		0.0090	0.0110	0.0210	0.0277	0.0223	0.0271
*S. cruzi*	0.1999	0.1973	0.1911	0.1835	0.1887	0.0984	0.0978	0.0985		0.0100	0.0210	0.0277	0.0205	0.0269
*S. capreolicanis*	0.1939	0.1905	0.1850	0.1842	0.1657	0.0864	0.0916	0.1003	0.1015		0.0216	0.0269	0.0251	0.0289
*S. taeniata*	0.1874	0.1877	0.1925	0.1875	0.1780	0.1892	0.1848	0.1820	0.2097	0.1822		0.0255	0.0238	0.0276
*S. tarandivulpes*	0.2085	0.2108	0.2281	0.2211	0.2184	0.2131	0.2121	0.2108	0.2251	0.2217	0.2247		0.0229	0.0385
*S. capracanis*	NA	NA	NA	NA	NA	NA	NA	NA	NA	NA	NA	NA		0.0313
*S. arieticanis*	NA	NA	NA	NA	NA	NA	NA	NA	NA	NA	NA	NA	NA	

*N* Norwegian isolates, *P* Polish isolates, *NA* not available

### Molecular characterization of the *ssu rRNA* gene

We obtained eight sequences and compared them with the sequences stored in the GenBank. All the sequences were eventually identified as *S. tenella* and submitted to the GenBank under the accession numbers shown in Table 1.

Similar as in the case of the *cox1* gene, the *ssu rRNA* sequences from Polish Tatra chamois were compared with the available *S. tenella ssu rRNA* gene sequences (GenBank Accession No. KC209734–KC209737). All the GenBank sequences were obtained from sheep sarcocysts from Norway. The *ssu rRNA* sequences isolated from Polish specimens contained ten polymorphic sites as compared to only one polymorphic site in the Norwegian isolates (Table S3). Three sheep sequences (KC209734–KC209736) and one sequence from Tatra chamois (KP263759) turned out to be identical. The average number of pairwise differences and nucleotide diversity in the sequences from Tatra chamois were approximately sevenfolds higher compared to the sheep sequences (Table 2). The analysis at a haplotype level revealed that one out of the two haplotypes from sheep isolates was identical with the haplotype from Tatra chamois (Fig. S3).

The phylogenetic relationships between the *ssu rRNA* genes of *Sarcocystis* spp. specimens isolated from cervids, cattle, and sheep were analyzed on the basis of ML, MP, and Bayesian inference. While the trees obtained with the three methods generally showed similar topologies, two principal differences were documented. Firstly, we found high inter-method variance in branching for the clade consisting of *S. tarandi*, *S. truncata*, *S*. sp., *S. rangiferi*, *S. elongata*, and *Sarcocystis silva*, and none of the methods was able to group the sequences of the same species together. As the problem was reported previously (Gjerde 2013) and did not directly influence our findings, we eventually did not include this part of the tree in further analyses. Secondly, the three methods used for the phylogenetic reconstruction produced different results regarding the relationship between *S. tenella* and *Sarcocystis capracanis*. While only one *S. tenella ssu rRNA* sequence (KP263755) clustered together in with the sequence from *S. capracanis* (L76472) in the MP and ML trees, the latter sequence was clustered with all the *S. tenella* sequences in the tree constructed with the Bayesian inference.

## Discussion

*Sarcocystis* spp. are common parasites of cervids, cattle, and sheep. *S. tenella* was characterized well as a parasite of sheep and is an established etiological factor of enzootic muscle tissue parasitoses and neurological disorders, mainly in lambs. In this study, we used light microscopy, *cox1* and *ssu rRNA* gene sequencing, genetic population analysis, and phylogenetic inference to identify and characterize *S. tenella* isolated from Polish Tatra chamois. To the best of our knowledge, this was the first study to identify *Sarcocystis* spp. from Tatra chamois at a molecular level.

Although we examined various tissues of three adult Tatra chamois, all the eight sarcocysts were isolated solely from the diaphragm and latissimus dorsi muscle specimens. In contrast, the authors of previous studies involving sheep (Pomroy and Charleston 1987; Dubey et al. 1988; Oryan et al. 1996) isolated *S. tenella* also from all other tissues that have been examined during the course of our experiment. Morphology of all the isolated cysts matched with the previously described characteristics of *S. tenella* cysts from sheep, obtained up to 60 days postinfection with this parasite (Mehlhorn et al. 1975). Older *S. tenella* cysts isolated from sheep were shown to present with fine palisade-like protrusions (Mehlhorn et al. 1975), not observed in the case of our sarcocysts from Tatra chamois. To the best of our knowledge, only two cases of *Sarcocystis* spp. invasion in Alpine chamois have been reported thus far (Boch and Schneidawind 1988; Odening et al. 1996). Boch and Schneidawind described three *Sarcocystis* types in Alpine chamois, including one identified morphologically as *S. tenella*. In turn, Odening et al. (1996) isolated two morphological types of *Sarcocystis*, which they referred to as *S*. sp. and *Sarcocystis cornagliai*.

Although *S. tenella* was demonstrated to cause a severe disease in sheep (Munday et al. 1975; Caldow et al. 2000; Schock et al. 2012; Agerholm and Dubey 2014), we cannot speculate on a potential pathogenicity of this species in Tatra chamois due to a small number of examined cadaveric specimens and the fact that we found only few microscopic cysts in the analyzed tissues.

Based on the analysis of *cox1* and *ssu rRNA* gene sequences from the eight sarcocysts isolated from Polish Tatra chamois, we identified them as the cysts of *S. tenella*. To this date, this parasite was isolated exclusively from sheep. The comparative analysis of our *cox1* gene sequences from Tatra chamois and the sequences from sheep sarcocysts described by Gjerde (2013) revealed that the latter presented with a higher number of both overall and private substitutions. In contrast, our *ssu rRNA* sequences from Tatra chamois contained more substitutions than the sheep sequences. Such high variability of the *ssu rRNA* sequences from Polish *S. tenella* is rather surprising as our previous study of other *Sarcocystis* spp. (Kolenda et al. 2014) revealed that this gene is highly conservative among the isolates from a single population. Previous research on *Eimeria* and *Sarcocystis* spp. (Ogedengbe et al. 2011; Gjerde 2014a; Kolenda et al. 2014) showed that *cox1* gene provides higher resolution of interspecies genetic differences than *ssu rRNA*. We obtained similar results during the comparative analysis of *S. tenella* and other closely related taxa (Table 3).

One aim of our study was to verify if *cox1* and/or *ssu rRNA* genes can be used as molecular markers for phylogeographic studies of *S. tenella*. Therefore, we compared the sequences of these genes from Polish and Norwegian specimens at a haplotype level (Figs. S2 and S3) and subjected them to a phylogenetic reconstruction (Fig. 1). All the Polish *cox1* sequences could be separated from their Norwegian counterparts at a haplotype level. Unfortunately, the phylogenetic analysis revealed that the differences between the Polish and Norwegian sequences were too subtle to discriminate their geographic origin from an evolutionary tree. Furthermore, the two groups of sequences could not be distinguished on the basis of mean pairwise distance matrix (Table 3). Also, the results for *ssu rRNA* sequences were not satisfactory. One haplotype from Tatra chamois turned out to be identical with the sheep haplotype, and we were unable to distinguish between the sequences with different geographic origin on the basis of their phylogenetic reconstruction. Moreover, the evolutionary trees obtained with various methods branched *S. capracanis* with *S. tenella* isolates in a different manner. Poor performance of evolutionary trees for the *ssu rRNA* gene sequences was previously reported by Gjerde. According to this author, this likely resulted from the difficulties in aligning hypervariable regions of *ssu rRNA* sequences between different *Sarcocystistidae* species (Gjerde 2014a). The analysis of mean pairwise distance matrix for the *ssu rRNA* sequences confirmed the results obtained during genetic population and phylogenetic analyses (Table 3). Due to lack of sequences from *S. tenella* isolated from sheep living in the area inhabited by Tatra chamois and poor performance of *cox1* and/or *ssu rRNA* genes as molecular markers for phylogeographic studies, we were unable to conclude if the minor sequence differences between Tatra chamois and Norwegian sheep isolates reflected the presence of country-specific variants or rather differences between the hosts.

Our findings provide a novel insight into intermediate host range for *S. tenella*. While *S. tenella* was only reported in sheep thus far (Mehlhorn et al. 1975; Haziroglu et al. 2002; Rassouli et al. 2014), we showed that also wildlife animals may be a reservoir of this parasite. Both sheep and chamois are members of the Caprinae subfamily, which implies that also other species from this group are susceptible to *S. tenella* infection. Our findings are consistent with the results of several recent studies of game animals, which have also found that the same *Sarcocystis* sp. might occur in more than one host (Dahlgren and Gjerde 2007, 2010; Gjerde 2014b). Previous studies showed that final hosts for *S. tenella* are canids (Dubey et al. 1982), and probably dogs and foxes are final hosts for this parasite in the region of Tatra mountains. Still, more work should be done to confirm life cycle stages and transmission of this pathogen between intermediate hosts and final hosts in Tatra mountains.

In summary, this is the first study to demonstrate that Tatra chamois can be an intermediate host for *S. tenella*. Our findings suggest that *cox1* and *ssu rRNA* genes can be used as genetic markers for identification of *S. tenella*, with *cox1* gene providing better resolution during phylogenetic analyses. Furthermore, we showed that *cox1* and *ssu rRNA* genes do not perform well as molecular markers for phylogeographic studies of *S. tenella*.

## Electronic supplementary material

Fig. S1 Light microscopic appearance of *Sarcocystis tenella* (200x) (JPEG 282 kb)

Fig. S2 Inter-haplotypic distance matrix for *cox1* gene. Heat map shows differences between the haplotypes of *cox1* gene. GenBank accession numbers of one representative strain from each haplotype are displayed on the x and y axes. None of the haplotypes represents more than one sequence. A color key containing histogram is presented with the chart. “Value” on the x axes and the color gradient correspond to the number of nucleotide differences between two haplotypes. “Count” on the y axes describes the number of squares with occurring value. (GIF 78 kb)

High resolution image (TIFF 184 kb)

Fig. S3 Inter-haplotypic distance matrix for *ssu rRNA* gene. Heat map shows differences between the haplotypes of *ssu rRNA* gene. GenBank accession numbers of one representative strain from each haplotype are displayed on the x and y axes. Two haplotypes are represented by more than one sequence (i.e., KP263754 and KP263758; KP263759 and KC209734–KC209736). A color key containing histogram is presented with the chart. “Value” on the x axes and the color gradient correspond to the number of nucleotide differences between two haplotypes. “Count” on the y axes describes the number of squares with occurring value. (GIF 54 kb)

High resolution image (TIFF 116 kb)

Table S1 (DOCX 19 kb)

Table S2 (DOCX 18 kb)

Table S3 (DOCX 14 kb)
