# Supplementary figures and images for: First molecular characterization of Sarcocystis tenella in Tatra chamois (Rupicapra rupicapra tatrica) in Poland
Source: Parasitol Res. 2015 Jul 24;114(10):3885–92. doi: 10.1007/s00436-015-4619-4 (PMC4561999; doi:10.1007/s00436-015-4619-4)

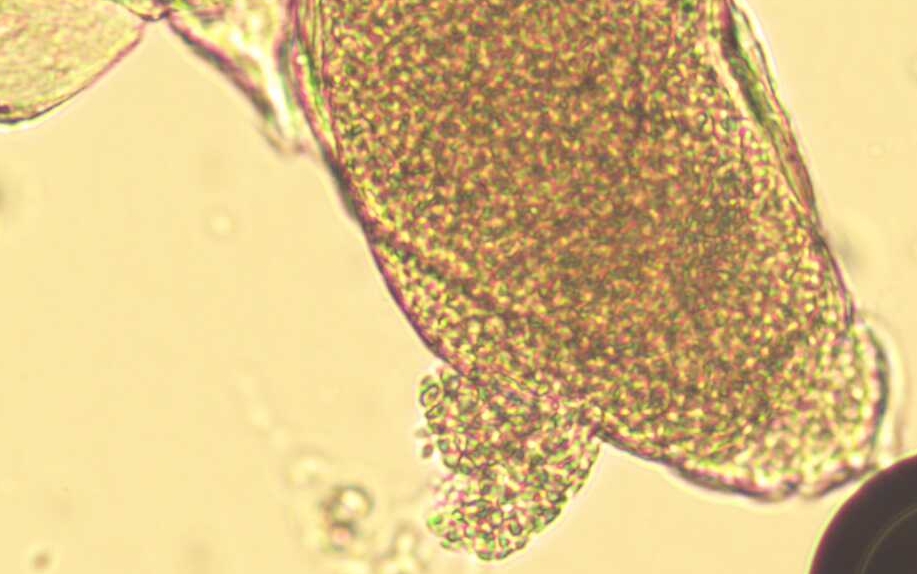

Supplement: Supplementary file 1 — Light microscopic appearance of Sarcocystis tenella (200x) (JPEG 282 kb) [file 436_2015_4619_Fig2_ESM.jpg]

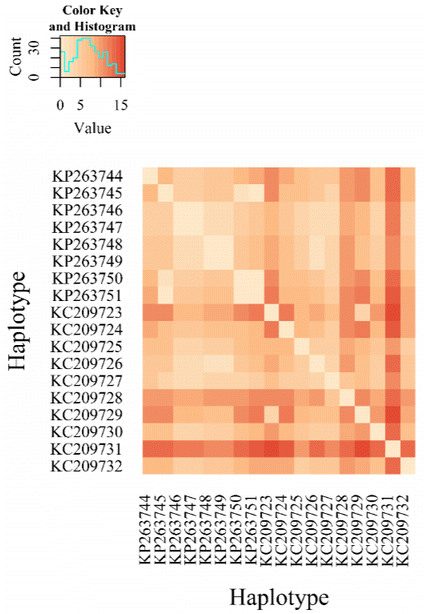

Supplement: Supplementary file 2 — Inter-haplotypic distance matrix for cox1 gene. Heat map shows differences between the haplotypes of cox1 gene. GenBank accession numbers of one representative strain from each haplotype are displayed on the x and y axes. None of the haplotypes represents more than one sequence. A color key containing histogram is presented with the chart. “Value” on the x axes and the color gradient correspond to the number of nucleotide differences between two haplotypes. “Count” on the y axes describes the number of squares with occurring value. (GIF 78 kb) [file 436_2015_4619_Fig3_ESM.gif]

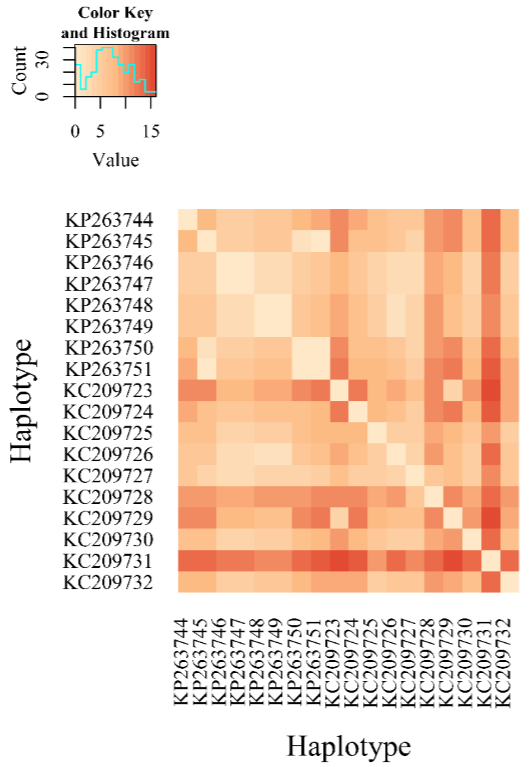

Supplement: Supplementary file 3 — High resolution image (TIFF 184 kb) [file 436_2015_4619_MOESM1_ESM.tif]

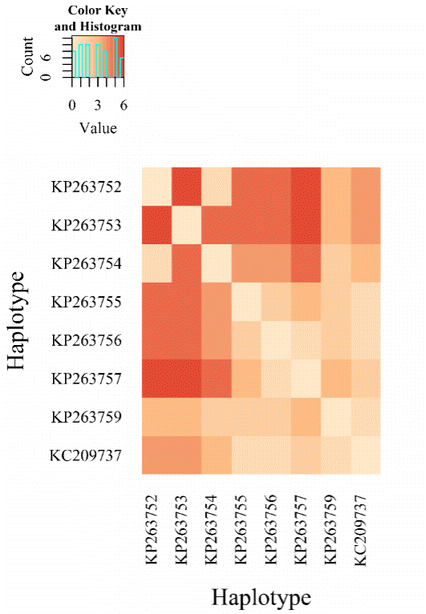

Supplement: Supplementary file 4 — Inter-haplotypic distance matrix for ssu rRNA gene. Heat map shows differences between the haplotypes of ssu rRNA gene. GenBank accession numbers of one representative strain from each haplotype are displayed on the x and y axes. Two haplotypes are represented by more than one sequence (i.e., KP263754 and KP263758; KP263759 and KC209734–KC209736). A color key containing histogram is presented with the chart. “Value” on the x axes and the color gradient correspond to the number of nucleotide differences between two haplotypes. “Count” on the y axes describes the number of squares with occurring value. (GIF 54 kb) [file 436_2015_4619_Fig4_ESM.gif]

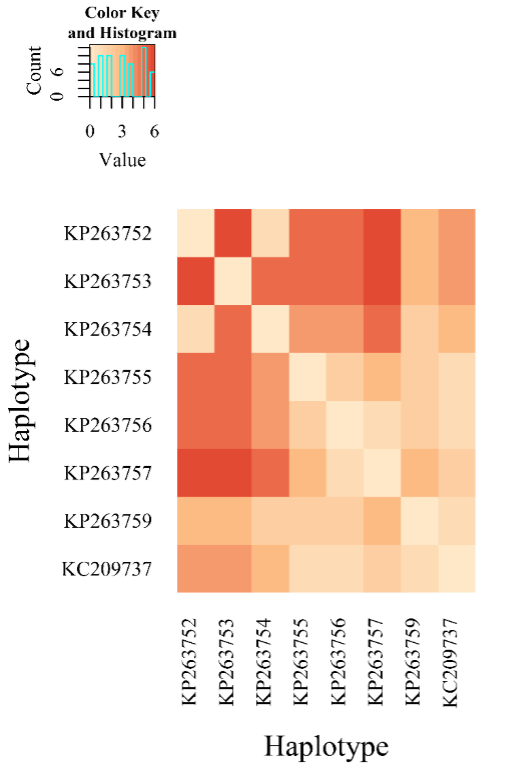

Supplement: Supplementary file 5 — High resolution image (TIFF 116 kb) [file 436_2015_4619_MOESM2_ESM.tif]
