# Supplementary material for: First molecular characterization of Sarcocystis tenella in Tatra chamois (Rupicapra rupicapra tatrica) in Poland
Source: Parasitol Res. 2015 Jul 24;114(10):3885–92. doi: 10.1007/s00436-015-4619-4 (PMC4561999; doi:10.1007/s00436-015-4619-4)
Supplement: Supplementary file 6 — (DOCX 19 kb) [file 436_2015_4619_MOESM3_ESM.docx]

First molecular characterization of *Sarcocystis* *tenella* in Tatra chamois (*Rupicapra rupicapra tatrica*) in Poland

Rafał Kolenda^1,*^, Peter Schierack^1^, Filip Zieba^2^, Tomasz Zwijacz-Kozica^2^, Michał Bednarski^3,**^

Brandenburg University of Technology Cottbus-Senftenberg, Faculty of Natural Sciences, Großenhainer Str. 57, D-01968, Senftenberg, Germany^1^

Tatra National Park, Kuźnice 1, 34-500 Zakopane ^2^

Department of Epizootiology and Clinic of Bird and Exotic Animals , Wrocław University of Environmental and Life Sciences, 50-375 Wrocław, Poland^3^

* Corresponding author at: Faculty of Natural Sciences, Brandenburg University of Technology Cottbus-Senftenberg, Großenhainer Str.57, D-01968 Senftenberg, Germany; Fax: +49 357385809, E-mail: rafal.kolenda@hs-lausitz.de

** Corresponding author at: Department of Epizootiology and Clinic of Bird and Exotic Animals, Wrocław University of Environmental and Life Sciences, 50-375 Wrocław, Poland; Fax: +48 713205336; E-mail: michal.bednarski@up.wroc.pl

**Table S1.** GenBank accession numbers of *cox1* and *ssu rRNA* gene sequences used for phylogenetic analysis.

| Species | GenBank Accession Nos. | |
| --- | --- | --- |
|  | *cox1* | *ssu rRNA* |
| *S. tenella* | KC209723- KC209732, KP263744- KP263751 | KC209734- KC209737, KP263752- KP263759, |
| *S. alces* | KC209578-KC209587, KF241309- KF241310,  KF831244- KF831247 | EU282018, KF831273- KF831274 |
| *S. alceslatrans* | KC209588- KC209593,  KF831248- KF831251 | EU282033, KF831275- KF831276 |
| *S. capreolicanis* | KC209594- KC209596, KF241311 | JN226117- JN226119 |
| *S. cruzi* | KC209597- KC209600 | AB682779- AB682780, JX679467, KC209738- KC209740 |
| *S. gigantea* | KC209601- KC209604 | KC209733, L24384 |
| *S. gracilis* | KC209605- KC209614, KF898100- KF898106, KF241330- KF241341 | KF880741, FJ196261 |
| *S. grueneri* | KC209615- KC209624 | EF056010 |
| *S. hardangeri* | KC209625- KC209633 | GQ250987, EF056014, EF467654 |
| *S. hirsuta* | KC209634 | KC209741, AF017122 |
| *S. hjorti* | KC209635- KC209643, KF241342- KF241354 | GQ250990, EU282017, KF831294- KF831295 |
| *S. ovalis* | KC209644- KC209655, KF241355- KF241382 | GQ250988- GQ250989, EU282019, EU282034 |
| *S. oviformis* | KC209656- KC209661, KF898107- KF898109 | KC209745- KC209746, KF880742, FJ196262 |
| *S. rangi* | KC209662- KC209670 | EF056011, EF467655, |
| *S. rangiferi* | KC209671- KC209676, KF241383- KF241409 | GQ250977- GQ250986, EF056015- EF056016 |
| *S. truncata* | KC209677- KC209683, KF241439- KF241452 | GQ251021- GQ251030 |
| *S. scandinavica* | KC209684- KC209685 | EU282020- EU282032 |
| *S. silva* | KC209686- KC209689, KF898110- KF898113, KF241410 | EU282016, JN226122- JN226125, KF880743 |
| *S. sinensis* | KC209690- KC209696 | JX679466, JX679469, KC209742- KC209744 |
| *S. hominis* |  | JX679470- JX679471 |
| *S. tarandi* | KC209697- KC209704, KF241411- KF241438 | GQ250967- GQ250976, EF056017- EF056018 |
| *S. elongata* | KC209705-KC209711, KF241312- KF241329 | GQ251011- GQ251020 |
| *S. tarandivulpes* | KC209712- KC209722 | EF056012, EF467656- EF467657 |
| *S. rileyi* | KJ396582 | KJ396583 |
| *S. taeniata* | KF831252- KF831272 | KF831277- KF831293 |
| *S. arctica* | KF601318- KF601325 | KF601301-KF601305 |
| *S. lutrae* | KF601326- KF601327 |  |
| *S. calchasi* |  | GQ245670 |
| *S. lacertae* |  | AY015113 |
| *S. buffalonis* |  | AF017121 |
| *S. fusiformis* |  | U03071 |
| *S. neurona* |  | U07812 |
| *S. capracanis* |  | L76472 |
| *S. moulei* |  | L76473 |
| *S. arieticanis* |  | L24382 |
| *S. muris* |  | M64244 |
| *Sarcocystis* sp*.* |  | AB251926, AB257085- AB257086, AB257154- AB257162 |
| *E. mitis* | JN864949 | U40262 |
| *E. acervulina* | HQ702479 | U67115 |
| *E. brunetti* | HQ702480 | U67116 |
| *E. maxima* | HQ702481 | DQ538348 |
| *E. necatrix* | HQ702482 | U67119 |
| *E. praecox* | HQ702483 | U67120 |
| *E. tenella* | HQ702484 | U67121 |
| *H. triffittae* | JX473247- JX473249 | GQ984222 |
| *H. heydorni* | JX473250- JX473251 | GQ984224 |
| *H. hammondi* |  | AF096498 |
| *N. caninum* | JX473252 | U16159 |
| *T. gondii* | JX473253 | EF472967 |
| *I. orlovi* |  | AY365026 |
| *I. belli* |  | DQ060683 |
| *B. besnoiti* |  | AF109678 |
| *B. jellisoni* |  | AF291426 |
