# Supplementary material for: First molecular characterization of Sarcocystis tenella in Tatra chamois (Rupicapra rupicapra tatrica) in Poland
Source: Parasitol Res. 2015 Jul 24;114(10):3885–92. doi: 10.1007/s00436-015-4619-4 (PMC4561999; doi:10.1007/s00436-015-4619-4)
Supplement: Supplementary file 7 — (DOCX 18 kb) [file 436_2015_4619_MOESM4_ESM.docx]

First molecular characterization of *Sarcocystis* *tenella* in Tatra chamois (*Rupicapra rupicapra tatrica*) in Poland

Rafał Kolenda^1,*^, Peter Schierack^1^, Filip Zieba^2^, Tomasz Zwijacz-Kozica^2^, Michał Bednarski^3,**^

Brandenburg University of Technology Cottbus-Senftenberg, Faculty of Natural Sciences, Großenhainer Str. 57, D-01968, Senftenberg, Germany^1^

Tatra National Park, Kuźnice 1, 34-500 Zakopane ^2^

Department of Epizootiology and Clinic of Bird and Exotic Animals , Wrocław University of Environmental and Life Sciences, 50-375 Wrocław, Poland^3^

* Corresponding author at: Faculty of Natural Sciences, Brandenburg University of Technology Cottbus-Senftenberg, Großenhainer Str.57, D-01968 Senftenberg, Germany; Fax: +49 357385809, E-mail: rafal.kolenda@hs-lausitz.de

** Corresponding author at: Department of Epizootiology and Clinic of Bird and Exotic Animals, Wrocław University of Environmental and Life Sciences, 50-375 Wrocław, Poland; Fax: +48 713205336; E-mail: michal.bednarski@up.wroc.pl

Table S2. Comparison of polymorphic sites with the *cox1* gene from *S. tenella*

|  | 4  5 | 5  6 | 1  3  8 | 1  4  6 | 1  7  1 | 2  1  1 | 2  1  3 | 3  6  7 | 4  1  1 | 4  7  4 | 4  7  7 | 4  7  8 | 4  8  4 | 5  1  3 | 5  4  1 | 5  4  6 | 5  8  8 | 6  0  0 | 6  1  3 | 6  2  2 | 6  2  6 | 6  4  8 | 6  5  1 | 6  5  7 | 6  8  0 | 6  9  0 | 7  1  1 | 7  4  7 | 8  0  7 | 8  2  2 | 8  6  4 | 8  9  1 | 9  0  9 | 9  1  5 | 9  3  0 | 9  3  4 | 9  3  5 | 9  4  4 | 9  5  7 | 9  7  2 | 9  9  0 |
| --- | --- | --- | --- | --- | --- | --- | --- | --- | --- | --- | --- | --- | --- | --- | --- | --- | --- | --- | --- | --- | --- | --- | --- | --- | --- | --- | --- | --- | --- | --- | --- | --- | --- | --- | --- | --- | --- | --- | --- | --- | --- |
| KP263744 | A | T | C | T | A | C | C | G | A | G | C | G | G | C | T | C | T | A | T | G | C | G | T | T | C | T | C | C | C | A | C | C | C | C | T | G | T | A | A | C | T |
| KP263745 | . | . | . | . | G | A | . | . | . | . | . | . | . | . | . | . | C | . | . | . | . | . | . | . | T | . | . | . | . | . | . | . | . | A | . | . | C | . | G | T | . |
| KP263746 | . | . | . | . | G | . | T | . | . | . | . | . | . | . | . | . | C | . | . | . | . | . | . | . | . | . | . | . | . | . | . | . | . | . | . | . | . | . | G | T | . |
| KP263747 | . | . | . | . | G | . | T | . | . | . | . | . | . | . | . | . | C | . | . | . | . | . | . | . | . | . | . | . | . | . | . | . | . | . | . | . | . | . | G | T | . |
| KP263748 | . | . | . | . | G | . | . | . | . | . | . | . | . | . | . | . | C | . | . | . | . | . | . | C | . | . | . | . | T | . | . | . | . | . | . | . | . | . | G | T | . |
| KP263749 | . | . | . | . | G | . | . | . | . | . | . | . | . | . | . | . | C | . | . | . | . | . | . | C | . | . | . | . | T | . | . | . | . | . | . | . | . | . | G | T | . |
| KP263750 | . | C | . | . | G | A | . | . | . | . | . | . | . | . | . | . | C | . | . | . | . | . | . | . | . | . | . | . | . | . | . | . | . | A | . | . | C | . | G | T | . |
| KP263751 | . | C | . | . | G | A | . | . | . | . | . | . | . | . | . | . | C | . | . | . | . | . | . | . | T | . | . | . | . | . | . | . | . | A | . | . | C | . | G | T | . |
| KC209732 | . | . | . | . | G | . | . | . | . | . | . | . | . | . | . | A | C | . | . | . | T | . | . | . | . | . | . | . | . | . | . | . | . | . | C | T | . | . | G | T | . |
| KC209731 | . | . | T | G | G | . | . | . | . | . | . | . | . | . | . | A | . | G | . | . | . | . | . | . | . | . | T | G | T | T | G | . | . | A | . | . | . | . | G | T | . |
| KC209730 | . | . | . | . | G | . | . | . | . | A | . | . | . | . | . | . | C | . | . | . | . | . | . | . | . | . | . | A | . | . | . | . | . | . | . | . | . | . | G | T | C |
| KC209729 | . | . | . | . | G | . | . | . | . | . | . | . | . | T | C | A | C | . | C | . | . | . | . | C | . | C | . | . | . | . | . | . | A | . | . | . | . | . | G | T | . |
| KC209728 | . | . | . | . | G | . | . | . | G | . | . | T | . | . | . | A | . | . | . | . | . | . | . | . | . | C | . | . | . | . | . | T | . | A | . | . | . | . | G | T | C |
| KC209727 | . | . | . | . | G | . | . | . | . | . | . | . | . | . | . | . | C | . | . | . | . | . | . | . | . | C | . | . | . | . | . | . | . | A | . | . | . | . | G | T | . |
| KC209726 | . | . | . | . | G | . | . | . | . | . | . | . | . | . | . | . | C | . | . | . | . | . | . | C | . | . | . | . | . | . | . | . | . | . | . | . | . | C | G | T | . |
| KC209725 | . | . | . | . | G | . | . | . | . | . | . | . | . | . | . | A | C | G | . | . | . | . | C | . | . | . | . | . | . | . | . | . | . | . | . | . | . | . | G | T | . |
| KC209724 | G | . | . | . | G | . | . | A | . | . | . | . | . | . | . | . | C | . | . | A | . | A | . | . | . | . | . | . | . | . | . | . | . | A | . | . | . | . | G | T | . |
| KC209723 | . | . | . | . | G | . | . | . | . | . | A | . | C | . | C | A | C | . | C | . | . | . | . | . | . | C | . | . | . | . | . | . | A | . | . | . | . | . | G | T | . |
