# Supplementary material for: First molecular characterization of Sarcocystis tenella in Tatra chamois (Rupicapra rupicapra tatrica) in Poland
Source: Parasitol Res. 2015 Jul 24;114(10):3885–92. doi: 10.1007/s00436-015-4619-4 (PMC4561999; doi:10.1007/s00436-015-4619-4)
Supplement: Supplementary file 8 — (DOCX 14 kb) [file 436_2015_4619_MOESM5_ESM.docx]

First molecular characterization of *Sarcocystis* *tenella* in Tatra chamois (*Rupicapra rupicapra tatrica*) in Poland

Rafał Kolenda^1,*^, Peter Schierack^1^, Filip Zieba^2^, Tomasz Zwijacz-Kozica^2^, Michał Bednarski^3,**^

Brandenburg University of Technology Cottbus-Senftenberg, Faculty of Natural Sciences, Großenhainer Str. 57, D-01968, Senftenberg, Germany^1^

Tatra National Park, Kuźnice 1, 34-500 Zakopane ^2^

Department of Epizootiology and Clinic of Bird and Exotic Animals , Wrocław University of Environmental and Life Sciences, 50-375 Wrocław, Poland^3^

* Corresponding author at: Faculty of Natural Sciences, Brandenburg University of Technology Cottbus-Senftenberg, Großenhainer Str.57, D-01968 Senftenberg, Germany; Fax: +49 357385809, E-mail: rafal.kolenda@hs-lausitz.de

** Corresponding author at: Department of Epizootiology and Clinic of Bird and Exotic Animals, Wrocław University of Environmental and Life Sciences, 50-375 Wrocław, Poland; Fax: +48 713205336; E-mail: michal.bednarski@up.wroc.pl

Table S3. Comparison of polymorphic sites with the *ssu rRNA* gene from *S. tenella*

|  | 1  4  5 | 1  5  8 | 2  1  0 | 5  6  5 | 6  9  4 | 7  1  4 | 7  2  4 | 7  4  5 | 7  5  7 | 1  5  7  8 |
| --- | --- | --- | --- | --- | --- | --- | --- | --- | --- | --- |
|  |  |  |  |  |  |  |  |  |  |  |
|  |  |  |  |  |  |  |  |  |  |  |
|  |  |  |  |  |  |  |  |  |  |  |
| KP263752 | G | C | G | C | T | A | T | C | A | G |
| KP263753 | A | T | . | . | . | G | . | T | G | A |
| KP263754 | A | . | . | . | . | . | . | . | . | . |
| KP263755 | A | . | A | . | . | G | C | T | . | . |
| KP263756 | A | . | . | . | C | G | C | T | . | . |
| KP263757 | A | . | . | T | C | G | C | T | . | . |
| KP263758 | A | . | . | . | . | . | . | . | . | . |
| KP263759 | A | . | . | . | . | G | . | T | . | . |
| KC209734 | A | . | . | . | . | G | . | T | . | . |
| KC209735 | A | . | . | . | . | G | . | T | . | . |
| KC209736 | A | . | . | . | . | G | . | T | . | . |
| KC209737 | A | . | . | . | . | G | C | T | . | . |
